# Wind-Related Orientation Patterns in Diurnal, Crepuscular and Nocturnal High-Altitude Insect Migrants

**DOI:** 10.3389/fnbeh.2016.00032

**Published:** 2016-02-29

**Authors:** Gao Hu, Ka Sing Lim, Don R. Reynolds, Andy M. Reynolds, Jason W. Chapman

**Affiliations:** ^1^Department of Agroecology, Rothamsted ResearchHarpenden, UK; ^2^College of Plant Protection, Nanjing Agricultural UniversityNanjing, China; ^3^Natural Resources Institute, University of GreenwichChatham, UK; ^4^Environment and Sustainability Institute, University of ExeterCornwall, UK

**Keywords:** entomological radar, insect migration, flight altitude, orientation cues, flight behavior, atmospheric turbulence, insect vision

## Abstract

Most insect migrants fly at considerable altitudes (hundreds of meters above the ground) where they utilize fast-flowing winds to achieve rapid and comparatively long-distance transport. The nocturnal aerial migrant fauna has been well studied with entomological radars, and many studies have demonstrated that flight orientations are frequently grouped around a common direction in a range of nocturnal insect migrants. Common orientation typically occurs close to the downwind direction (thus ensuring that a large component of the insects’ self-powered speed is directed downstream), and in nocturnal insects at least, the downwind headings are seemingly maintained by direct detection of wind-related turbulent cues. Despite being far more abundant and speciose, the day-flying windborne migrant fauna has been much less studied by radar; thus the frequency of wind-related common orientation patterns and the sensory mechanisms involved in their formation remain to be established. Here, we analyze a large dataset of >600,000 radar-detected “medium-sized” windborne insect migrants (body mass from 10 to 70 mg), flying hundreds of meters above southern UK, during the afternoon, in the period around sunset, and in the middle of the night. We found that wind-related common orientation was almost ubiquitous during the day (present in 97% of all “migration events” analyzed), and was also frequent at sunset (85%) and at night (81%). Headings were systematically offset to the right of the flow at night-time (as predicted from the use of turbulence cues for flow assessment), but there was no directional bias in the offsets during the day or at sunset. Orientation “performance” significantly increased with increasing flight altitude throughout the day and night. We conclude by discussing sensory mechanisms which most likely play a role in the selection and maintenance of wind-related flight headings.

## Introduction

From virtually the first moments that special-purpose entomological scanning radars were deployed in the late-1960s (Schaefer, 1976), observers were astonished to note that moderate and large-sized *nocturnal* insect migrants, flying independently of each other at high altitudes, often showed flight headings (i.e., body orientations, not displacement directions) which were tightly grouped about some common direction, and that the common orientation may remain constant for periods of several hours. This was a surprise because it had previously been assumed that, under the often very low illumination (starlight) conditions experienced, there would be a lack of environmental cues to enable large numbers of high-flying insects to consistently maintain the same orientation. In addition, it had been assumed (erroneously) that winds at flight altitudes would so dominate the migrants’ displacements, that there would be little point in them taking up any particular headings as they would be carried downwind irrespective of their self-powered flight direction. Over the succeeding decades, however, persistent “common orientation” has been observed in many different species under diverse environmental conditions, in many areas of the world, and particularly at night (Chapman et al., 2011a; Drake and Reynolds, 2012).

The fact that the migrants do not select a common orientation direction by visual reference to each other—they are generally too widely spaced for that (Riley, 1989)—clearly indicates that the individuals involved respond to the same environmental cue (or cues) in approximately the same manner. Frequent observations of the *broad-scale* nature of the common orientation phenomenon, often extending over hundreds or even thousands of square kilometers (Hobbs and Wolf, 1996; Lang et al., 2004; Rennie et al., 2010; Rennie, 2014), demonstrates that common orientation is not a response to local topographical features such as river valleys, a ridge of hills, or roads. So, what is the nature of the environmental cue (or cues) that is responsible for huge numbers of high-flying insects taking up and maintaining the same flight heading over very large spatial scales?

Our recent studies of the common orientation patterns of nocturnal insects, carried out with the new generation of autonomously-operating vertical-looking radars (VLRs; Chapman et al., 2011a; Drake and Reynolds, 2012), have shown that high-flying nocturnal insects generally take up flight headings which are close to the downwind direction. There are clear benefits associated with downwind orientation, as the insect’s self-powered flight vector (typically 1–5 m/s) will be added to the downwind vector (typically 5–20 m/s at flight heights), resulting in maximum tailwind assistance and rapid displacement (Chapman et al., 2011b, 2016). In some cases, downwind orientation seemingly occurs irrespective of the wind direction which will result in a random distribution of displacements over the course of a season (representing a kind of “dispersal”), while in others downwind orientation is combined with selection of favorable tailwinds to achieve rapid long-range migration to seasonally-beneficial habitat zones (Chapman et al., 2015b). The latter case is exemplified by the in-depth study of the nocturnal migratory flights of the silver-Y moth (*Autographa gamma*) in Europe. These studies have demonstrated that the moths only migrate on nights with seasonally-beneficial winds (towards the north in the spring and towards the south in the autumn), that they select the altitude with the fastest winds, and that they orientate close to the downwind direction so that they achieve maximum displacement in a beneficial direction (Chapman et al., 2010), resulting in significant reproductive benefits for the migrants (Chapman et al., 2012).

It is clear that, at least for the well-studied nocturnal insects, the downwind direction is the principal environmental feature to which the migrating insects attempt to orientate, and that this results in broad-scale (wind-related) common orientation of multiple species. But how do the insects detect the downwind direction while flying within the moving current hundreds of meters above the ground? One possibility is that they identify their displacement direction by visual assessment of the apparent motion of ground features, but for nocturnal insects there is considerable evidence that this is not the case (Riley and Reynolds, 1986; Reynolds et al., 2010). Another possible mechanism, which now has good support for nocturnal migrants, is that the insects identify the downwind direction by detecting and responding to micro-turbulence cues which are postulated to be stronger in the flow direction (Reynolds et al., 2010; Aralimarad et al., 2011; Chapman et al., 2015a). A quirk of the postulated micro-turbulence mechanism is that insects which attempt to align themselves with the flow direction are predicted to have a systematic bias in their mean heading direction relative to the flow when the Ekman spiral is present (Reynolds et al., 2010). This effect will result in headings being slightly offset consistently to the *right* of the flow direction in the northern hemisphere (and to the left in the southern hemisphere) whenever the Ekman spiral is present (typically in the stable atmospheric conditions present at night), and this prediction can be tested if large datasets are available.

While there is good evidence that *nocturnal* insects migrating over the UK routinely demonstrate a high degree of common orientation closely aligned with the downwind direction, but typically offset to the *right* of the flow (Aralimarad et al., 2011; Chapman et al., 2015a) as expected from the theory (Reynolds et al., 2010), patterns of wind-related orientation in the considerably more diverse and abundant *diurnal* aerial insect fauna have not been studied at all. In this article, we analyze a large dataset of >600,000 radar-detected “medium-sized” windborne insect migrants (body mass from 10 to 70 mg), migrating during the afternoon, in the period around sunset, and in the middle of the night, detected at three VLR sites in southern England during spring, summer and autumn over a 10 year period. This selected mass range is significantly smaller than the well-studied *A. gamma* moths (which weigh ~100–200 mg) referred to above, and so this (and similar) noctuid moth species will be excluded from the data analyzed. We reveal for the first time the frequency of common orientation of flight headings in these medium-sized day-flying insects, and the association of their heading distributions with the downwind direction, and discuss the implications of these findings for the likely sensory modality responsible for the patterns observed.

## Materials and Methods

### The Vertical-Looking Radar (VLR)

We used data collected by two fully-automated, vertical-looking X-band radars (VLRs), incorporating a narrow-angle conical scan and rotating linear polarization, located at sites in southern England. One VLR has operated at Rothamsted Research, Harpenden, Hertfordshire (lat. 51° 48′ 32″ N, long. 0° 21′ 27″ W) since 1999, while the other was at Malvern, Worcestershire (lat. 52° 06′ 04″ N, long. 2° 18′ 38″ W) from 2000 to 2003, and then at Chilbolton, Hampshire (lat. 51° 8′ 40″ N, long. 1° 26′ 13″ W) from 2004 onwards. Data is acquired simultaneously from 15 height bands (each 45 m deep) arranged between ~150 and 1200 m above the radar, during 5-min sampling periods, repeated every 15 min, day and night. If the insect targets are individually-resolvable they are usually well described by our underlying analysis model, and the following parameters can be routinely extracted: displacement speed (relative to ground), displacement direction or track (relative to ground), body alignment (the flight heading, but with a 180° ambiguity), and three radar scattering cross-section terms from which the target’s mass and shape can be estimated (for further technical details, see Chapman et al., 2002).

### Data Extraction

A new method of data extraction and analysis using the *R* software environment (version 3.1.2^1^) allowed us to be ambitious in the data mining of our large VLR database and in fact, we analyzed directional data from >600,000 individual insects from a 10 year span (2000–2009) of continuous operation of both radars, divided into representative daytime (14.00–17.00 GMT), crepuscular (120 min centered around the time of sunset), and night-time (22.00–00.00 GMT) periods. We initially selected all “medium-sized” insects (with a body mass of 10–70 mg) detected by the radars across all 15 altitude ranges (~150–1200 m) in each of the three time periods during the months of May to September 2000–2009. This resulted in a total of 811,316 insects from 7815 different occasions (i.e., a date/radar site/time period combination during which at least one insect was detected) being abstracted from our database (Table 1). The data were further divided into the following seasonal periods: “Spring” (May–June), “Summer” (July), and “Autumn” (August–September). In order to remove occasions with small sample sizes of individual insect targets, we restricted analysis to a subset of occasions which together comprised 75% of the total number of insects in each of the time period/seasonal period categories. This reduced the number of occasions analyzed to 1869 “migration events” (comprising 24% of the total), which nonetheless encompassed 75% of the total number (608,223) of insects (Table 1).

**Table 1:** **Orientation characteristics of “migration events”**.

Period	Season	No. of events		No. of insects		Common orientation (%)	Heading *r* value
		All	Selected	All	Selected		(Mean ± SE)
Daytime	Spring	986	182	126,883	95,159	93.41 (170/182)	0.366 ± 0.012
	Summer	584	163	228,568	171,376	98.16 (160/163)	0.424 ± 0.014
	Autumn	1104	234	197,394	147,987	99.15 (232/234)	0.525 ± 0.011
	Subtotal	2674	579	552,845	414,522	97.06 (562/579)	0.448 ± 0.007
Sunset	Spring	1020	299	61,985	46,482	75.25 (225/299)	0.339 ± 0.009
	Summer	589	169	60,702	45,486	91.12 (154/169)	0.425 ± 0.014
	Autumn	1138	330	75,132	56,290	90.61 (299/330)	0.469 ± 0.009
	Subtotal	2747	798	197,819	148,258	84.96 (678/798)	0.416 ± 0.006
Night	Spring	766	133	12,841	9628	72.93 (97/133)	0.470 ± 0.014
	Summer	558	125	18,996	14,212	76.80 (96/125)	0.376 ± 0.014
	Autumn	1070	234	28,815	21,603	87.18 (204/234)	0.485 ± 0.011
	Subtotal	2394	492	60,652	45,443	80.69 (397/492)	0.455 ± 0.008
Total		7815	1869	811,316	608,223

### Circular Statistics

The VLRs automatically record the displacement direction (the movement direction relative to the ground) and the body alignment of every individual insect. The body alignment represents the flight heading of the insect (i.e., the direction in which the insect would fly in the absence of wind), albeit with a 180° ambiguity as it is not possible to distinguish the head-end from the tail-end from the radar signal alone. However, extensive analysis in previous studies have demonstrated that of the two possible values from the body alignment, the true flight heading is the one which is closest to the insect’s displacement direction (which at flight altitudes will usually be close to the downwind direction) and so that value is selected to represent the true heading (Chapman et al., 2008, 2010, 2013, 2015a; Aralimarad et al., 2011; Drake and Reynolds, 2012). We then used the Rayleigh test of uniformity for circular data to calculate the mean displacement direction, and the mean flight heading, for each of the 1869 “migration events”. Circular statistics associated with the calculation of the mean flight heading were also produced: *r* (a measure of the clustering of the angular distribution of headings ranging from 0 to 1, with higher values indicating tighter clustering and thus a greater degree of common orientation around the mean) and the probability that the distribution of headings differed from a uniform distribution. If the Rayleigh test *p*-value was <0.05, this indicated that the distribution of headings during that particular “migration event” was significantly unimodal (i.e., statistically oriented in the same direction), and thus that event was characterized as exhibiting a significant degree of common orientation. Conversely, if the Rayleigh test *p*-value was >0.05, then the distribution of headings during that particular “migration event” was characterized as not exhibiting common orientation.

Comparisons of the proportion of “migration events” exhibiting significant common orientation (i.e., the proportion with Rayleigh *p*-values <0.05), and the magnitude of the offsets of the mean heading from the wind flow, were then made between time periods and seasons. Orientation “performance” during different seasons and time-periods was assessed by comparing the mean heading *r*-values (a measure of the tightness of common orientation) with ANOVAs and *t*-tests, as *r*-values are inversely related to the degree of scatter of individuals around the mean and thus represent a measure of how well the aerial fauna in each event align their heading with the flow. In addition, the effect of flight altitude on the tightness of common orientation was investigated by calculating the heading *r*-value separately at each altitude range whenever there were >20 insects at that height during each of the “migration events”, and then carrying out a linear regression of heading *r*-value on flight altitude. Finally, to examine the size and direction (left or right) of the offset of the mean heading from the flow during the “migration events”, we calculated the magnitude of the difference between the mean heading and the mean displacement direction (a proxy for the flow), and the direction of this offset relative to the displacement, in each event. Left offsets were assigned negative values (because they do not match the expectations of the turbulence mechanism; Reynolds et al., 2010), while right offsets were assigned positive values (because they do match the expectations). We then analyzed the distribution of these offsets to see if there was a significant bias towards one side of the flow or not, for each time period and season separately, by calculating the mean offset and 95% confidence intervals and comparing these with an expected mean offset of zero (which would be expected if either the turbulence mechanism was not used to align with the flow, or if the Ekman spiral was not present).

## Results

**Figure 1:**
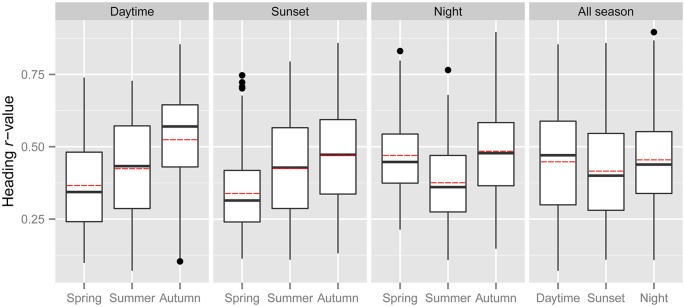
**The distribution of heading *r*-values of “migration events” during the spring, summer and autumn of the three time periods (three panels on the left), and for all seasons combined during the three time periods (panel on the right).** The bottom and top of the box show the lower and upper quartile values, respectively. The horizontal solid black line represents the median for each category, and the red dashed line represents the mean. Whiskers indicate the 5th and 95th percentiles, while the black circles show the outliers.

Common orientation in medium-sized insect migrants (i.e., occasions when the distribution of flight headings was significantly unimodal) was frequent in all time periods, varying from 73–99% of the “migration events” depending upon time period and season (Table 1). It was most frequent during the daytime (afternoon) period, with an overall mean frequency of 97% across all seasons, followed by the sunset period (85%) and then the night-time period (81%; Table 1). When seasons were combined, orientation “performance” (i.e., the magnitude of heading *r*-values, see “Materials and Methods” Section) was significantly affected by time period (ANOVA: *F* = 8.9, *df* = 21,634, *p* < 0.0001; Table 2; Figure 1); pairwise *t*-tests showed that *r*-values were significantly higher during the daytime and night-time periods than during the sunset period (*p* < 0.001 for both pairwise comparisons), but *r*-values during day and night were not significantly different (*p* = 0.54; Table 3; Figure 1). There was also a seasonal effect on orientation “performance” within each of the three time-periods (ANOVA: *p* < 0.0001 in each case; Table 2; Figure 1). Orientation “performance” significantly improved (i.e., heading *r*-values increased) with the progression from spring to autumn for the daytime and sunset periods (Figure 1), but the pattern was more complex during the night-time when values were lowest in the summer. All possible pairwise comparisons within a time-period, with one exception (spring vs. autumn during night-time), were significantly different from each other (*t*-tests: *p* < 0.01 in each case; Table 3).

**Table 2:** **ANOVA results for heading *r*-value against season and time period**.

Period	Factor	*df*	*F*	*p*
Daytime	Season	2559	47.649	<0.0001
Sunset	Season	2675	46.983	<0.0001
Night	Season	2394	17.782	<0.0001
All period	Period	21,634	8.889	<0.0001

**Table 3:** ***t*-test results for heading *r*-value against season**.

Period	Group 1	Group 2	*t*	*df*	*p*
Daytime	Spring	Summer	−3.237	319	0.0013
	Spring	Autumn	−9.816	381	<0.0001
	Summer	Autumn	−5.737	337	<0.0001
Sunset	Spring	Summer	−5.292	269	<0.0001
	Spring	Autumn	−10.323	519	<0.0001
	Summer	Autumn	−2.680	291	0.0078
Night	Spring	Summer	4.666	191	<0.0001
	Spring	Autumn	−0.804	212	0.4223
	Summer	Autumn	−5.982	208	<0.0001
All period	Daytime	Sunset	3.292	1154	0.0010
	Daytime	Night	−0.612	912	0.5405
	Sunset	Night	−3.862	858	0.0001

**Figure 2:**
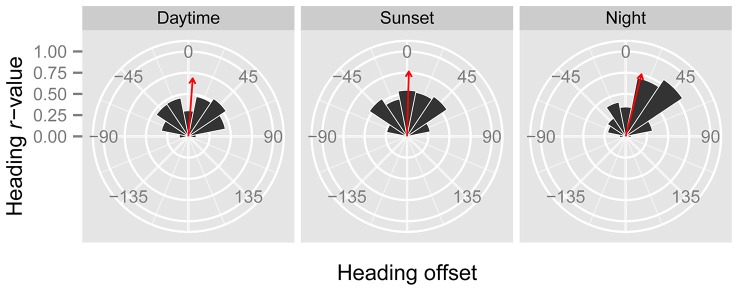
**Circular histograms of the offsets between the mean headings and the mean displacements of “migration events” during the three time periods.** Heading offsets to the right of the displacement have a positive value, while heading offsets to the left of the displacement have a negative value. The area of the black segments is proportional to the number of occasions when offsets fell within each 22.5° bin. The bearing of the red arrow indicates the mean offset of the entire dataset, while its length is proportional to the clustering of the dataset around the mean.

**Figure 3:**
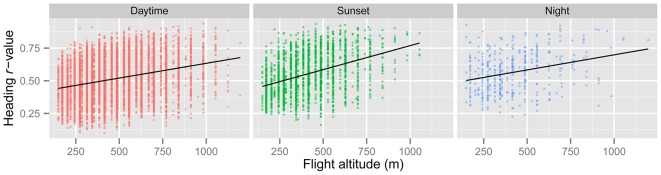
**Regression of *r* (a measure of angular dispersion around the mean flight heading) against insect flight altitude for the “migration events” during the three time periods.** Higher values of *r* denote occasions with greater orientation “performance” (i.e., tighter orientation around the mean). There is a highly significant positive relationship between *r* and flight altitude in all three periods (see Table 5), indicating that insects orient their flight headings with respect to the downwind direction better at higher altitudes.

In line with previous analyses (Reynolds et al., 2010; Aralimarad et al., 2011; Chapman et al., 2015a), our extensive analysis of the *nocturnal* migrants confirmed the highly significant, systematic offset of headings to the right of the flow direction. When data from all seasons were combined, there were many more “migration events” when the mean heading was to the right of the displacement direction (*n* = 262, 66%) compared to the left of the displacement (*n* = 135, 34%), and the overall mean offset was significantly different from zero (mean offset: +11.9°, 95% CI: +9.8°, +18.7°, *S* = 6.21, *p* < 0.0001; Table 4; Figure 2). In contrast to this clear right-hand bias at night, the split of headings on the right and left was almost equal during both the daytime (right: *n* = 298, 53%; left: *n* = 264, 47%) and at sunset (right: *n* = 363, 54%; left: *n* = 315, 46%). Neither of these distributions was significantly different from zero as the 95% CI included zero (daytime: +3.5°, 95% CI: −0.4°, +9.0°, *S* = 1.79, *p* = 0.073; sunset: +1.2°, 95% CI: −1.8°, +5.0°, *S* = 0.93, *p* = 0.353; Table 4; Figure 2). Finally, there were similar significant relationships between flight altitude and orientation “performance” in all three time periods, with heading *r*-values tending to increase with flight altitude in a linear fashion (Table 5; Figure 3). This indicates that insects were able to align themselves with the flow direction with greater accuracy as their flight altitude increased irrespective of the ambient illumination.

**Table 4:** **Offsets between heading and displacement direction of “migration events”**.

Periods	Season	Right: Left		95% CI	*S*	*p*
Daytime	Spring	58:112	−12.94	−23.14, −9.08	−4.4433	<0.0001
	Summer	96:64	10.06	4.40, 25.14	2.7749	0.0055
	Autumn	144:88	10.91	6.74, 18.92	4.0998	<0.0001
	Subtotal	298:264	3.45	−0.40, 8.97	1.7940	0.0728
Sunset	Spring	102:123	−6.17	−11.44, −1.54	−2.5651	0.0103
	Summer	88:66	3.96	−2.02, 13.96	1.4661	0.1426
	Autumn	173:126	5.22	0.72, 11.42	2.2216	0.0263
	Subtotal	363:315	1.16	−1.80, 5.05	0.9294	0.3527
Night	Spring	57:40	5.05	−1.88, 13.80	1.4911	0.1359
	Summer	56:40	8.47	−1.20, 24.24	1.7785	0.0753
	Autumn	149:55	16.71	13.86, 24.75	6.8271	<0.0001
	Subtotal	262:135	11.87	9.80, 18.71	6.2092	<0.0001

**Table 5:** **Regression of heading *r*-value against flight altitude**.

Period	*df*	*F*	*r*^2^	*p*
Daytime	15,807	504.40	0.080	<0.0001
Sunset	12,014	376.10	0.157	<0.0001
Night	1397	36.38	0.082	<0.0001
Total	18,222	724.90	0.081	<0.0001

## Discussion

This study presents the first large-scale and systematic analysis of the wind-related orientation behavior of both day-flying and night-flying insects, using a large dataset of radar-detected high-flying insect migrants. The size range of the radar-detected insects was restricted to so-called “medium-sized” insects (with a body mass of 10–70 mg), for two reasons. The upper size limit was selected to exclude the large species of Lepidoptera, such as the nocturnal noctuid moths *A. gamma* and *Noctua pronuba*, and the day-flying butterflies *Vanessa cardui* and *V. atalanta*, which have been studied in depth previously (Chapman et al., 2008, 2010, 2012, 2015a). The lower size limit was selected so that the smallest insects in our dataset were detectable over the majority of the vertical range of the VLRs (from 150 to 1200 m). It is not possible to fully characterize the aerial fauna detected by the VLRs in this size range, but from extensive aerial sampling campaigns carried out in the UK during the period analyzed (Chapman et al., 2004), we can identify some of the likely major constituents of each time period. Major components of the day-flying fauna in this size class are likely to include heteropteran bugs, hoverflies, ladybirds and carabid beetles (Chapman et al., 2005; Jeffries et al., 2013; Reynolds et al., 2013). Insects detected during the twilight period around sunset will include some relatively short-flying crepuscular species such as green lacewings and various nematoceran Diptera (Chapman et al., 2004, 2006), plus nocturnal species which takeoff at this time. Later in the night, the fauna will be dominated by longer-flying nocturnal species, including smaller Lepidoptera and various Coleoptera (Chapman et al., 2004). However, it is not possible to classify all of the radar targets analyzed in this study, and it is very likely that all of the “migration events” included a high diversity of species, especially during the daytime. Given the taxonomic diversity of insects involved, the consistency of the orientation patterns observed are therefore all the more surprising, and indicate that wind-related orientation is virtually ubiquitous among high-flying insect migrants of VLR-detectable size (i.e., with a body mass >10 mg).

Our results support the earlier demonstration that wind-related common orientation is frequent in nocturnal migrants in the UK (Aralimarad et al., 2011), but they also demonstrate for the first time that it is a more frequent (in fact almost universal) phenomenon during the daytime, occurring during 93–99% of the “migration events” in the size-range investigated (Table 1). The ability of migrants to achieve wind-related common orientation is seemingly similar during the day and night, as the mean heading *r*-value (our measure of orientation “performance”) was not significantly different between these two time periods. Common orientation was also frequent during the twilight period around sunset (75–91% of “migration events”), occurring with a similar frequency as during the night (73–87%). However, our measure of orientation “performance” was lower during the sunset period than either the day or night (Figure 1), suggesting that insects were less able to align themselves with the wind during this time period. This could be explained by the fact that many of the crepuscular insects are relatively short-range migrants which fly for just an hour or so, and which consequently invest less in orientation capabilities than the longer-flying daytime and night-time migrants, but this needs further research. Another possibility is that crepuscular migrants rely on visual cues to sense the downwind direction rather than turbulence-related cues (see below), and consequently suffer impaired orientation capabilities due to decreasing light levels compared to the day.

Previous studies have provided convincing evidence that nocturnal migrants use turbulence cues to identify and align themselves with the downwind direction, as they have consistently found a systematic bias in heading offsets to the right of the flow, as expected from the turbulence theory (Reynolds et al., 2010; Aralimarad et al., 2011; Chapman et al., 2015a); this finding was confirmed with a very extensive dataset in the present study. The right offsets are predicted due to the presence of the Ekman spiral, which is most common in the stable atmospheric conditions expected in fair-weather conditions at night. The presence of right offsets (in the northern hemisphere) has been postulated to be a so-called “smoking gun” for the turbulence mechanism of flow detection, because it is not clear what other mechanism could possibly lead to a systematic right-hand bias. However, it is perhaps (just) conceivable that right offsets may be an unsuspected consequence of a visual-based mechanism for assessing the flow direction. Analysis of heading offsets during day-flying and crepuscular insects allows this idea to be tested, as the Ekman spiral is much less likely to be present in the convective daytime atmosphere than it is in the stable night-time atmosphere. Our finding that heading offsets are not significantly different from zero (i.e., they are not systematically biased to either the left or the right of the flow) in either the daytime or the sunset period therefore provides additional evidence that nocturnal insects use turbulence cues for flow detection, as if right offsets were a consequence of a visually-mediated mechanism they would be expected to occur day and night. Conversely, the absence of right offsets during the daytime and sunset periods does not rule out a role for turbulence cues in the assessment of flow direction, as the Ekman spiral is typically not present in convective daytime atmospheres.

In order to gauge which mechanism(s), turbulence or visual, is most likely to be responsible for the frequent wind-related common orientation during the daytime and sunset periods, additional analyses are required. Under relatively stable atmospheric conditions (during the day and night), the turbulence theory predicts that the signal to noise ratio will increase with increasing height, and consequently common orientation may be expected to be tighter with increasing height. This prediction fits well with our observation that the angular dispersion of orientation significantly decreased with increasing flight altitude (Figure 3), in all time periods, which may indicate that the turbulence mechanism is also used during the day. However, there may be other reasons for this increase in orientation “performance” with height; for example, the visual cues required to assess displacement direction relative to the ground may possibly be easier to detect with increasing height due to the granularity of the landscape features involved. The observation that orientation “performance” tended to increase with the seasonal progression from “Spring” (May and June) through “Summer” (July) to “Autumn” (August and September) during the daytime and sunset periods may also suggest that the turbulence mechanism is not used during daylight hours. This is because thermal convection is expected to increase from spring to late-summer, and increased thermals will disrupt the postulated turbulence cues (which are only present in stable atmospheres; Reynolds et al., 2010). However, there is no reason to expect a seasonal progression in orientation “performance” if vision were the primary mechanism for assessing the flow direction during the day, and so for the moment there is no clear consensus on the orientation mechanism employed by day-flying migrants.

In Conclusion, this study demonstrates that wind-related common orientation is ubiquitous throughout the day and night in medium-sized insects across a wide range of taxa and this behavior will result in greater displacement speeds and increased migration distances. The persistent offsets of headings to the right of the flow provides further evidence that the nocturnal insects principally rely on turbulence cues to identify the flow direction. Day-flying and crepuscular migrants also exhibit common orientation, but the mechanism by which they identify the flow direction is unclear. The possibility exists that they may exclusively use the turbulence mechanism to identify the flow directly, exclusively use a visual mechanism to assess their displacement relative to the ground, or a combination of both, and further research is required to resolve this. The benefits of wind-related orientation include faster transport speeds, and thus an increase in the distance traveled compared to the energy expended during flight. These benefits will be greatest if migration is predominantly in seasonally-beneficial directions as we have found in the larger nocturnal migrants (Chapman et al., 2008, 2010, 2016); analysis of the seasonal and annual variability of the migration directions of the relatively under-studied day-flying fauna are ongoing and will be published separately (Hu and Chapman, unpublished data).

## Author Contributions

JWC designed the analysis, and GH performed the analysis with input from KSL. All authors contributed to the interpretation of results and manuscript writing.

## Conflict of Interest Statement

The authors declare that the research was conducted in the absence of any commercial or financial relationships that could be construed as a potential conflict of interest.

## Footnotes

^1^https://www.r-project.org/
